# The Interplay between Antibiotics and the Host Immune Response in Sepsis: From Basic Mechanisms to Clinical Considerations: A Comprehensive Narrative Review

**DOI:** 10.3390/antibiotics13050406

**Published:** 2024-04-28

**Authors:** Martina Tosi, Irene Coloretti, Marianna Meschiari, Sara De Biasi, Massimo Girardis, Stefano Busani

**Affiliations:** 1 Anesthesia and Intensive Care Medicine, Policlinico di Modena, University of Modena and Reggio Emilia, 41124 Modena, Italy; tosimartina@gmail.com (M.T.); irenecoloretti@gmail.com (I.C.); massimo.girardis@unimore.it (M.G.); 2 Infectious Diseases Unit, Policlinico di Modena, 41124 Modena, Italy; mariannameschiari1209@gmail.com; 3 Department of Medical and Surgical Sciences for Children & Adults, University of Modena, and Reggio Emilia, 41125 Modena, Italy; sdebiasi@unimore.it

**Keywords:** sepsis, immune response, antibiotic therapy, critically ill patients

## Abstract

Sepsis poses a significant global health challenge due to immune system dysregulation. This narrative review explores the complex relationship between antibiotics and the immune system, aiming to clarify the involved mechanisms and their clinical impacts. From pre-clinical studies, antibiotics exhibit various immunomodulatory effects, including the regulation of pro-inflammatory cytokine production, interaction with Toll-Like Receptors, modulation of the P38/Pmk-1 Pathway, inhibition of Matrix Metalloproteinases, blockade of nitric oxide synthase, and regulation of caspase-induced apoptosis. Additionally, antibiotic-induced alterations to the microbiome are associated with changes in systemic immunity, affecting cellular and humoral responses. The adjunctive use of antibiotics in sepsis patients, particularly macrolides, has attracted attention due to their immune-regulatory effects. However, there are limited data comparing different types of macrolides. More robust evidence comes from studies on community-acquired pneumonia, especially in severe cases with a hyper-inflammatory response. While studies on septic shock have shown mixed results regarding mortality rates and immune response modulation, conflicting findings are also observed with macrolides in acute respiratory distress syndrome. In conclusion, there is a pressing need to tailor antibiotic therapy based on the patient’s immune profile to optimize outcomes in sepsis management.

## 1. Introduction

Sepsis has a high global prevalence and accounts for approximately 20% of the global mortality burden [1]. The most severe forms escalate to septic shock, contributing to 10.4% of ICU admissions, with hospital mortality rates approaching 40% [2]. Several authors contend that the root causes of the most severe manifestations of sepsis lie in the emergence of abnormal immune reactions within the host’s body [3]. 

Two opposite pathophysiological models may well summarize the wide variety of immune response dysregulation occurring in sepsis. On the one hand, patients may develop an uncontrolled hyperinflammatory response with organ damage caused mainly by the mechanisms intended to neutralize pathogens; on the other hand, patients may present an unbalanced and prolonged anti-inflammatory response with failure of pathogen eradication (i.e., breakthrough infection) and occurrence of secondary infections [4]. The more the immune response is skewed toward one of the models, the higher will be the risk of death. In recent years, significant efforts have been made to identify additional treatments for sepsis aimed at modulating the host’s immune response, for instance intravenous IgM-enriched immunoglobulins [5], but none of them has shown a significant benefit yet. Appropriate antibiotic treatment along with source control remain the interventions with a real and proven impact on outcome [6]. Emerging evidence shows how, beyond their activity of microorganism killing and growth inhibition, antimicrobial drugs may also exert direct and indirect effects on the immune system. Given the diriment role of a balanced immune response during sepsis, the immunomodulatory effects of antibiotics must be understood and carefully considered during the selection of antimicrobial treatment in these patients.

This comprehensive narrative review aims to provide the latest findings on the immune-modulating proprieties of antibiotics, to describe the mechanisms involved and to discuss the possible clinical implications in septic patients. Most current studies retrieved are mainly pre-clinical investigations conducted on immune cell cultures, ex-vivo sepsis models utilizing harvested white blood cells, or animal studies. Only a few of the studies on the immunomodulatory effects of antimicrobial drugs have advanced to the stage of human clinical trials.

Given that the review’s objective is narrative rather than systematic, the bibliographic research for the manuscript was conducted on journals indexed on PubMed, albeit in a non-methodologically rigorous manner. Specifically, the following terms were employed for the different sections:

- For the section on mechanisms of action, terms such as ‘immune dysfunction’ and ‘sepsis pathophysiology’ were paired with each class of antibiotics, with exclusion criteria for papers related to clinical research, inflammatory/rheumatic diseases, cystic fibrosis, and chronic respiratory diseases.
- In the microbiome section, terms such as “antibiotics”, “immunomodulation/dysfunction”, “microbiota or microbiome”, and “sepsis” were utilized.
- Regarding the clinical studies section, the search terms included “antibiotics”, “immunomodulation/dysfunction”, and “clinical research”, with exclusion criteria for studies involving animals and in-vitro and ex-vivo methodologies. However, for each search, temporal criteria were restricted to the last 25 years of publications, from 1999 onwards.

## 2. Antibiotics and Immune Response: Mechanisms of Action

### 2.1. Regulation of Pro-Inflammatory Cytokines Production

Cytokines and chemokines are key regulators of the inflammatory response, orchestrating both proinflammatory (interleukin (IL)-1, IL-6, IL-8, tumour necrosis factor (TNF), and IFN-γ) and anti-inflammatory (e.g., IL-10) effects. Several studies have underscored the impact of antimicrobial drugs on the secretion of these mediators. Among the most well-documented immunomodulatory effects are those attributed to macrolides antibiotics, which diminish the production of pro-inflammatory cytokines (such as TNF, IL-1, IL-6, IL-8) through various mechanisms [7].

Intravenous administration of clarithromycin at therapeutic dosages has been shown to reduce serum concentration of TNF and reactive oxygen species in a murine model of sepsis caused by multi-sensitive *E. coli*, multidrug-resistant (MDR) *P. aeruginosa*, or pan-drug-resistant *K. pneumoniae*, and this was associated with survival improvement [8,9]. Furthermore, an earlier study demonstrated that clarithromycin could suppress the production of IL-8 stimulated by lipopolysaccharide (LPS) in human peripheral monocytes and human monocytic leukaemia cell line THP-1 [10]. Similarly, in a septic shock model, the oral administration of azithromycin reduced serum levels of TNF, which correlated with decreased lethality [11]. Azithromycin has also been proven to suppress IL-6, IL-10, IL-12 and TNF production in human monocyte-derived dendritic cells and CD4+ T cells [12]. Dey et al. reported that azithromycin was more effective than ciprofloxacin in regulating cytokine release from phagocytic cells [13]. In studies assessing cytokine production after stimulation with *staphylococcal* toxin, Kushiya et al. observed that azithromycin exhibited a slight suppression of pro-inflammatory cytokines production by toxin-stimulated peripheral blood mononuclear cells [14] (Table 1).

Beyond macrolides, Pichereau et al. investigated the effects of various antibiotics on cytokine production from peripheral blood mononuclear cells after exposure to *Staphylococcus aureus* toxin in an in-vitro study [15]. Antimicrobials with high tissue penetration, including tigecycline, clindamycin, trimethoprim/sulfamethoxazole, and linezolid suppressed cytokines production in a concentration-dependent manner, whereas this effect was not observed with vancomycin and daptomycin[13]. Furthermore, vancomycin appears to directly stimulate the production of anti-inflammatory mediators, as demonstrated by measurement of LPS-stimulated IL-10 release. Other studies indeed demonstrated a significant decrease in cytokine levels in septic patients treated with daptomycin [16,17]. Even quinolones appear to exert pronounced anti-inflammatory effects, as demonstrated in LPS-activated human mononuclear cells with lowering of Th1 and Th2 cytokine expression [18,19]. Following ciprofloxacin, profound unresponsiveness of immune-competent cells to LPS stimulation was observed in an ex-vivo model of sepsis [20] (Table 1). Again, fosfomycin seems to exert an anti-inflammatory effect due to the reduction of proinflammatory cytokine synthesis in mice via several mechanisms [21] (see below). 

Conversely, cephalosporins, as demonstrated by Ziegeler et al., appear to induce a shift from a balanced to an inflammatory cytokine release pattern. This study reported a decrease in LPS-stimulated IL-10 release from all cephalosporins, with a more pronounced effect observed with cefuroxime and cefotaxime [20]. Also, colistin seems to exert pro-inflammatory activities, as described by Wang et al., who reported a significant increase in the secretion of cytokines TNF, IL-1β, and IL-6 by stimulated macrophages in a murine model [22] (Table 1). 

### 2.2. Modulation of Toll-like Receptors (TLRs) and Regulation of P38/Pmk-1 Pathway

TLRs are primarily activated by bacterial pathogen-associated molecular patterns that elicit a comprehensive inflammatory response during infections. Due to their multifaceted roles, TLRs have been considered suitable target molecules for immune modulatory treatments in sepsis [23]. Several classes of antibiotics interact with the TLRs system. Among them, erythromycin, doxycycline, and moxifloxacin exhibit the most notable activity, demonstrating significant modulation in TLRs’ expression and regulation of monocyte phagocytic activity [24]. Linezolid, vancomycin, and daptomycin, which are largely used in critically ill patients, were investigated on the TRL pathway in sepsis models, too [25] (Table 1). LPS-activated THP-1 monocytes were incubated with the antibiotics and gene expression of various cytokines, and the TLRs were monitored. The results revealed an upregulation of the expression of all TLRs induced by linezolid and vancomycin. In contrast, daptomycin downregulated transcription of the TLRs responsible for the recognition of pathogen-associated molecular patterns from Gram-positive bacteria. The authors concluded that linezolid and, especially, vancomycin exhibit mainly pro-inflammatory effects, whereas daptomycin demonstrates anti-inflammatory properties under sepsis-like conditions. Moreover, cefuroxime and cefotaxime were also found to decrease the expression density of the LPS recognition molecule CD14 on monocytes, thus downregulating, in this way, monocytes’ response to LPS [20] (Table 1). Recent observations have examined the impact of ceftaroline on neutrophil function, including its effects on phagocytic response, expression of TLRs, cell survival, and apoptosis. This innate neutrophil function was detailed in healthy patients whose cells were exposed to lipoteichoic acid (LTA) of S. aureus, reporting that ceftaroline exerts immunostimulatory effects on phagocytosis higher than other antibiotics on neutrophils exposed to LTA [26]. Again, fosfomycin seems to exert an immunomodulatory effect by inhibiting TLR/NF-κb/MAPK signalling pathways. Fosfomycin further diminishes NLRP3 inflammasome activation. In a rat model of sepsis-induced lung injury, fosfomycin decreased the expression of TLR-4, NF-κb, and TNF in lung tissue. Fosfomycin also exhibited antioxidative properties in this setting [27].

The increasing prevalence of MDR pathogens has prompted a reassessment of older antibiotics such as colistin. Despite extensive research into its antibacterial properties, there is a scarcity of data regarding colistin’s impacts on immune pathways. An animal study revealed that a short, non-toxic treatment with colistin can activate the innate immunity of *C. elegans* via a p38/PMK-1-dependent pathway [28]. This pathway, crucial for innate immunity activation, appears to be conserved from plants to mammals [29]. Furthermore, it has been demonstrated that colistin can induce an upregulation of specific genes and transcriptional responses that closely resemble those triggered by microorganisms. A further study on rat macrophages observed that colistin enhances the secretion of pro-inflammatory cytokines and improves the macrophages’ phagocytic activity against *S. aureus* by modulating the p38/MAPK pathway in a dose-dependent manner [30] (Table 1). 

### 2.3. Inhibition of Matrix Metalloproteinases (MMPs) and Blockade of Nitric Oxide (NO) Synthase

MMPs are enzymes capable of degrading the extracellular matrix in various tissues, and their activity is heightened during inflammation processes. Clinical studies have linked elevated MMPs levels with tissue damage and increased mortality in septic patients [29,30,31]. In acute respiratory distress syndrome (ARDS), a correlation has been noted between MMP activation, illness severity, and outcome [32,33]. Although the precise mechanisms remain uncertain, it has been demonstrated that tetracyclines can diminish MMPs activity, thus potentially mitigating organ damage during hyperinflammation, particularly in the context of pulmonary infections. Hyperproduction of NO by the inducible form of NO synthase (iNOS) may contribute to the vasoplegia and cardio-depression observed in septic shock [34,35]. Inhibition of iNOS, either through translational or transcriptional mechanisms leading to reduced production of NO, has been reported for macrolides and tetracyclines [36]. Additionally, other antibiotics such as tigecycline and quinolones appear capable of inhibiting LPS-induced nitric oxide production [37,38]. English et al. observed that daptomycin exposure reduced the inflammatory response of macrophages to *S. aureus* (reduced TNF release and reduced the accumulation of iNOS compared to those with vancomycin or oxacillin) [39]. In an in-vitro rat model, polymyxin B was tested for its ability to reduce LPS-induced NO production [40]. Additionally, ceftazidime significantly decreased nitrosative stress by reducing the concentrations of pulmonary nitrotyrosine formation in sheep with acute lung injury and sepsis [41]. All these listed properties could potentially be valuable in clinical practice, particularly in septic shock with severe hyperinflammatory states for limiting vasodilation and restoring hemodynamics (Table 1).

### 2.4. Regulation of Apoptosis Induced by Caspases

The significance of apoptosis in lymphoid organs and parenchymal tissues has garnered heightened attention in the understanding of sepsis pathophysiology, particularly concerning organ and immune dysfunction [42]. Many studies have highlighted the pivotal role of the caspase pathway in apoptosis, pyroptosis, and necroptosis during infections, particularly in conditions of sepsis [43]. Indeed, both caspase inhibitors and caspase deficiency have shown significant improvements in survival and overall disease outcome in sepsis, and with protection from high-dose endotoxin shock and sepsis-related cardiac dysfunction in animal models [44,45]. A potential interaction between certain antibiotics and this apoptotic pathway has been observed in other disease models: for instance, minocycline has demonstrated protection against apoptosis by inhibiting caspases in various conditions such as ischemia-reperfusion injury and stroke [46,47], although there is a shortage of data on the effects of antimicrobial drugs on caspases in sepsis (Table 1). Azithromycin appears to enhance bacterial clearance and attenuate lung injury in mice chronically infected with Pseudomonas aeruginosa by inhibiting caspase-1-dependent IL-1ß and IL-18 secretion [48], as previously highlighted in our report on the inflammasome pathway in MDR pathogens [49]. A recent study examined the effects of tetracyclines on a murine model of acute lung injury (ALI) and inflammation. Tetracyclines were found to significantly reduce ALI and pulmonary infiltrations, primarily through the selective inhibition of caspase-1-dependent IL-1β and IL-18 production, ultimately leading to improved survival rates. Additional experiments demonstrated that tetracyclines also decrease the production of IL-1β and IL-18 by ex-vivo-studied alveolar leukocytes from patients with direct ARDS, suggesting a potential role for tetracyclines in the treatment of ARDS associated with elevated caspase-1 activity [50] (Table 1). 

## 3. Antibiotics and Sepsis Impact on Microbiome Disruption

The administration of broad-spectrum antibiotics disrupts the composition of the gut with a loss of commensal bacteria and an overgrowth of potential pathogenic microorganisms. These alterations also extend to nonbacterial intestinal inhabitants. Disruptions of these intestinal communities are associated with both an increased susceptibility to develop sepsis as well as a higher risk of adverse outcomes [51].

### 3.1. Animal Studies

Several investigations indicate a connection between the preservation of gut microbiome balance and the effectiveness of systemic immunity, encompassing both innate and adaptive cellular and molecular pathways. Signals emanating from the microbiome appear to impact not only the generation of neutrophil granulocytes in the bone marrow but also that of macrophages and monocytes [52,53]. Additional reports indicated a decrease in circulating neutrophil granulocytes, their precursors, and factors and cytokines essential for haematopoiesis stimulation in mice subjected to antibiotic administration or in germ-free mice [52]. In addition to the production of immunity cells, their functionality is affected as well: neutrophil cells’ extravasation capacity from the bloodstream to the site of infection is impaired together with the bacterial killing capacity of neutrophils and macrophages as a consequence of lower production of ROS [54,55]. Besides the Pattern Recognition Receptors pathway, there are many cytokines whose production seems to be altered in microbiota-depleted animal models: IFN-1 [56], TNF, IL-6 [55], IL-18, IL-1β, IFN-ϒ, and IL-17 [57]. Yet, another intriguing study demonstrated the significant influence of microbiome composition on the generation of adaptive immune cells, including T CD4+ and CD8+ lymphocytes, as well as specific antibodies following infection with the Influenza A respiratory virus [58]. In this study, mice who received a combined antibiotic therapy of ampicillin, vancomycin, metronidazole, and neomycin, as well as those who received only neomycin, had extremely pronounced immune-suppressive effects on cytotoxic T lymphocytes, whereas in mice treated with ampicillin, vancomycin, or metronidazole alone the observed effects were variable and of average entity.

Other mechanisms have been observed by Clarke et al., who reported a reduced clearance capacity of K. pneumoniae in the lungs of microbiota-depleted mice [55], while Schuijt et al. observed, in the same pre-clinical setting, a reduced phagocytosis capacity, increased systemic dissemination, inflammation, organ dysfunction, and higher mortality rates [59]. Similarly, Chen et al. showed increased mortality rates in microbiota-depleted mice with pneumonia sustained by *E. coli* infection [60].

### 3.2. Human Studies

Besides antibiotics, the development of sepsis itself can alter microbiota. Sun et al. evaluated the microbiome of septic patients showing a destruction of symbiotic flora [61]. Patients with a high burden of Bacteroides, especially *B. vulgatus*, had higher severity scores and longer stays in the intensive care unit (ICU). The alteration of the gut microbiome also seems to be involved in sepsis myocardial injury; the mechanisms by which gut microbiota affects myocardial health include small-chains fatty acids, cytokines, and mitochondrial damage [62,63]. Various clinical studies in critically ill patients suggest that alterations in the richness and diversity of the microbiome may result in dysregulation of systemic immune responses to invasive pathogens [53]. A recent study part of the Human Functional Genomics Project investigated 500 healthy volunteers as to how differences in the composition of microbiome communities may affect the production of cytokines in answer to microbial stimuli [64]. Patients enrolled in the study of Lankelma et al. highlighted how the disruption of the microbiome, consequent to the administration of broad-spectrum antibiotics, leads to a reduced capacity of systemic cellular response, in terms of ex-vivo production of TNF, IL-1β, and IL-6 after stimulation with LPS [65]. Dufour et al. demonstrated a pronounced reduction of serum immunoglobulin G titers as a consequence of administration of amoxicillin/clavulanate [66].

Shimizu and colleagues reported how a variation in the composition of the gut microbial community, particularly a decrease of anaerobic forced bacteria and an increase of pathogen microorganisms, is associated with the development of septic complications and superior mortality rates among patients with systemic inflammatory response syndrome (SIRS) [67]. Another interesting signal in this direction is provided by a sub-analysis of an American large-scale study that identified in hospitalized patients a correlation between some conditions known to cause dysbiosis and re-hospitalization for severe sepsis in the following 90 days. Namely, patients hospitalized for infective events and even more patients with *C. difficile* infection, compared to patients with non-infective diseases, had subsequent higher re-admission rates for severe sepsis but comparable re-admission rates for non-sepsis-related diseases [68]. A remarkable study conducted by Shimizu et al. on critically ill patients analysed the outcomes of 52 patients with SIRS by dividing them into three groups based on the composition pattern of their faecal microbiome. The phenotypes may be classed as either differentiated, meaning with richness of bacteria of different taxa; single, in which a specific microorganism predominated on the others; or depleted, in which a general lack of bacteria can be observed. The observed mortality in the three groups was, respectively, 6%, 52%, and 64% [69]. A recent study conducted on 71 mechanically ventilated patients demonstrated that the administration of broad-spectrum antibiotics and disease severity may be associated with gut dysbiosis in the ICU. The progression of gut dysbiosis occurring in these patients was associated with higher mortality [70].

Regrettably, comparative studies are scarce, with only a handful of exceptions in small-scale inquiries that would allow us to accurately outline the unique immune-modulating characteristics of different antibiotic compounds and their actual effects on the microbiome.

## 4. Clinical Studies on Antibiotics and Immunity

The majority of the trials available on the use of antibiotic drugs as adjunctive treatment in patients with sepsis are focused on the use of macrolides, drugs whose immune-regulatory effects are better known and whose use is already an established practice in the framework of chronic inflammatory diseases [71]. Data comparing different macrolides are largely lacking. Here, we delve into a comprehensive examination of investigations concerning various clinical scenarios.

### 4.1. Community-Acquired Pneumonia (CAP) and Ventilator-Associated Pneumonia (VAP)

Several studies have indicated an improvement in survival and other clinical outcomes associated with the adjunctive use of macrolide therapy in CAP [72]. Nie et al. conducted a meta-analysis published on the treatment of CAP, comparing β-lactam monotherapy with the addition of a macrolide [73]. The results consistently showed a superior effect on patients’ survival with the combination therapy, regardless of whether they were in an intensive or non-ICU setting and irrespective of the degree of severity of the pneumonia. Garin et al. conducted a randomized controlled trial (RCT) of non-inferiority involving 580 patients to compare β-lactam monotherapy with β-lactam–macrolide combination therapy for the treatment of moderately severe CAP [74]. In contrast with the previously cited studies, they observed a difference in the rate of achieving clinical stability at day 7 only among patients with more severe forms of CAP. A prospective study on 52 patients with non-responding CAP demonstrated that macrolide treatment significantly altered cytokines expression in both plasma and bronchoalveolar lavage (BAL). IL-6 and TNF levels in BAL fluid were significantly lower in patients treated with macrolide regimens, showing a tendency towards decreased IL-8 levels. Interestingly, lower IL-6 levels in BAL were confirmed even after excluding patients receiving concomitant inhaled or systemic corticosteroid treatment. Additionally, circulating interleukins 8 and 10 were notably lower in patients receiving macrolide regimens, with a trend towards decreased IL-6 levels. In the subgroup of patients without concomitant corticosteroid treatment, lower levels of IL-6, IL-8, and IL-10 in those on macrolide regimens were observed [75]. The benefits of using a macrolide-containing antimicrobial regimen for the treatment of CAP were once again reaffirmed by two large retrospective studies conducted by a Spanish group. They observed a reduction in 30-day mortality among patients who received macrolides, with a more pronounced effect seen in those with high levels of inflammation, as indicated by CRP levels > 15 mg/dL, and in those with Pneumococcal pneumonia. The reduction in 30-day mortality was even greater when both criteria were met. Importantly, this benefit was consistently observed irrespective of whether the causative strain was resistant to macrolides [76,77].

Very recently, Giamarellos-Borboulis and co-workers published the results of the ACCESS trial, a multicentre RCT enrolling 278 hospitalized patients with CAP who presented with SIRS, a Sequential Organ Failure Assessment (SOFA) score of two or higher, and procalcitonin titre above 0.25 ng/mL [78]. Patients were randomly assigned to receive oral placebo or oral clarithromycin. In the clarithromycin group, 68% of patients met a composite endpoint, compared to 38% in the placebo group. Interestingly, there was no observed difference in the odds of achieving this composite outcome between patients with and without microbiologically documented infection, whether with bacterial or non-bacterial pathogens. These differences were accompanied by significant modifications in the expression of pro- and anti-inflammatory cytokines and in the ability of circulating peripheral blood mononuclear cells (PBMCs) to produce TNF between the two groups. Patients who received clarithromycin appeared to experience a less pronounced immune-paralysis effect in response to infection, as evidenced by an improved capacity of circulating PBMCs to produce TNF, a decrease in circulating IL-10 levels, and a decrease in the IL-8 to IL-10 ratio. While these observations do not directly provide evidence, they indirectly suggest an effect of clarithromycin on immune function [78]. Additionally, some benefits were observed in viral pneumonia as well. In a RCT involving 48 children with respiratory failure caused by *respiratory syncytial virus* infection, treatment with high-dose azithromycin reduced the length of ventilatory support and oxygen therapy as well as the length of hospital stay. Those benefits appeared to be mediated by a reduction of MMP-9, TNF, IL-1β, and IL-10 levels [79].

Only a few reports have been published about VAP and macrolides; a secondary analysis of an older RCT trial explained the observed outcome benefit by reporting differences in the pattern of molecular response among patients who received clarithromycin: the use of clarithromycin resulted in the restoration of the balance between pro-inflammatory and anti-inflammatory mediators, evidenced by significantly lower TNF levels, higher IL-6 levels, a more balanced TNF/IL-10 ratio, and more efficient processes of antigen presentation and apoptosis [80]. Notably, these differences were more pronounced among patients who developed septic shock. These findings seem to witness clarithromycin’s ability to reverse immune suppression and endotoxin tolerance and accelerate the return to homoeostasis from immune suppression and maintenance of innate immune cell function against invading pathogens, although evidence is not as strong as those for CAP.

### 4.2. Sepsis, Septic Shock, and ARDS

A recent secondary analysis of the MARS (Molecular Assessment and Risk Stratification in Sepsis) study assessed the effects of low-dose erythromycin on 235 patients, with 470 patients serving as controls. The study found no significant differences in mortality rates up to day 90 between patients treated with low-dose erythromycin and the control group, with a matching hazard ratio (HR) of 0.89 (95% CI 0.64–1.24) and a weighted HR of 0.95 (95% CI 0.66–1.36). Additionally, there were no disparities observed in secondary clinical outcomes. The alteration in levels of host response biomarkers from admission to day 4 showed similar patterns between erythromycin-treated patients and controls [81]. A recent meta-analysis including three RCTs involving a total of 910 patients did not reveal an improvement in short-term outcomes in patients with sepsis treated with clarithromycin [82]. In the recent INCLASS trial, 110 patients with sepsis and moderate hypoxia, along with more than three SOFA points derived from systems other than respiratory function, were randomly assigned to receive either intravenous adjunctive treatment with clarithromycin or a placebo over 4 days. The trial encompassed patients with sepsis in critical conditions, as witnessed by the 90% of patients requiring vasopressors. The results showed no discrepancy in survival rates at both 28 and 90 days between the two treatment groups, although, notably, patients administered with clarithromycin exhibited a more than half reduction in the risk of developing secondary infections. Moreover, the subgroup analysis demonstrated a favourable impact on 90-day mortality among those with a SOFA score exceeding 12 at enrolment. These subgroups’ clinical benefits observed in the clarithromycin group could potentially be attributed to the modulation of the host immune response, aiding in the recovery from sepsis-induced immunosuppression. This is evidenced by the observed increase in mHLA-DR expression and expansion of non-classical monocytes in the cohort of patients who received clarithromycin [83]. 

In a comprehensive retrospective secondary analysis conducted by Simonis et al. [84], the impact of macrolide usage was evaluated in a cohort comprising 873 ARDS patients. The study found that patients who received macrolide therapy, primarily erythromycin, during their ICU stay experienced a decrease of nearly 10% in mortality at the 30-day mark. Upon further examination of the study population, it was observed that the survival advantage was evident predominantly in ARDS cases stemming from non-pulmonary causes. Notably, subgroup analysis unveiled a more significant benefit of macrolide exposure in ARDS cases exhibiting biological phenotype I, identified as the less inflamed type. This observation poses a challenge as it appears to contradict the presumed mechanism of action of macrolides [67].

## 5. Conclusions

**Figure 1:**
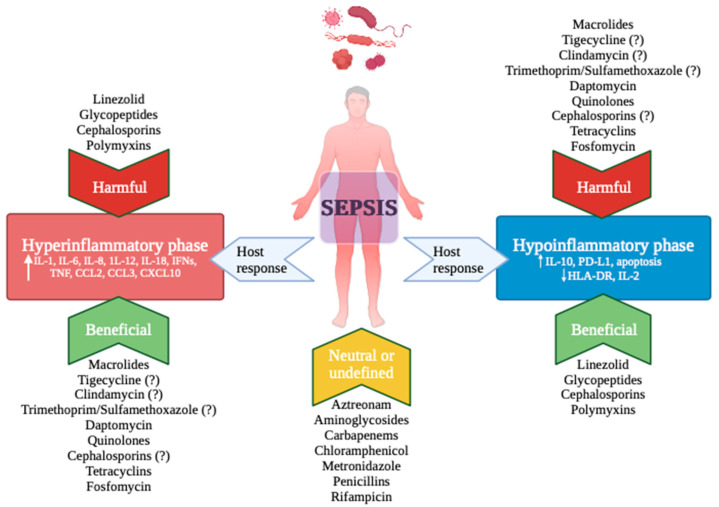
The figure depicts antibiotics that may have beneficial, detrimental, or neutral effects concerning the two phases of the host’s (hyper- or hypo) inflammatory response in sepsis. Legenda: IL: Interleukin, IFN: Interferon, TNF: Tumour Necrosis Factor, CCL: Chemokine Ligand, CXCL: C-X-C motif chemokine ligand.

We have entered a new phase to fight infections, one that focuses on boosting the interaction between sepsis and the immune system. We know that the inflammatory-immune response can vary greatly, influenced by factors such as microbial load, virulence, host genetics, and existing health conditions [3]. Given the diverse phenotypes seen in septic patients, customizing treatment approaches could be immensely beneficial. Antibiotics, being central to sepsis treatment, offer not only the means to fight the infection but also the potential to modulate the immune response (Figure 1). In this context, the selection of antibiotics should not solely depend on the source, location, and cause of the infection but also on the specific immune profile of the patient [85] (Figure 1). This idea gains support from research on macrolides, which have demonstrated the ability to dampen inflammation. The positive outcomes observed in patients with CAP could be attributed to considering the immunological state of these patient groups. Further basic and clinical investigations are imperative to delve deeper into this area, given the current limited evidence.

## Figures and Tables

**Table 1:** How antibiotics affect the immune response through different basic mechanisms of action (see text for details).

Antibiotic’s Class	Pro-Inflammatory Cytokines Production	TLRs Expression Modulation	MMPs and NO	Apoptosis Regulation
**Macrolides**	Suppression (*Clarithromycin*, *Azithromycin*)	Modulation in TLRs and control of phagocytic activity (*Erythromycin*).	Reduced production (*all macrolides*)	Suppression of caspases activity resulting in enhanced bacterial clearance (*Azithromycin*)
**Tigecycline**	Suppression in concentration-dependent manner		Inhibition of NO production induced by LPS	
**Tetracyclines**		Modulation in TLRs expression and regulation of phagocytic activity *(Doxycycline*)	Decreased MMPs activity, mitigating inflammation. Reduced NO production	Inhibition of caspases against apoptosis (*Minocycline*)
**Clindamycin**	Suppression in concentration-dependent manner			
**Trimethoprim** **-** **Sulfamethoxazole**	Suppression in concentration-dependent manner			
**Linezolid**	Suppression in concentration-dependent manner	Upregulation of TLRs expression resulting in pro-inflammatory effect		
**Glycopeptides**	Stimulation of anti-inflammatory cytokines (*Vancomycin*)	Upregulation of TLRs expression resulting in pro-inflammatory effect (*Vancomycin*)		
**Daptomycin**	Suppression	Downregulation of TLRs transcription resulting in anti-inflammatory effect	Reduction of accumulation	
**Quinolones**	Suppression (*Ciprofloxacin*)	Modulation in TLRs expression and regulation of phagocytic activity (*Moxifloxacin*)	Inhibition of NO production induced by LPS	
**Cephalosporins**	Stimulation (*Cefuroxime, Cefotaxime*)	Downregulation of TLRs transcription resulting in anti-inflammatory effect (*Cefuroxime, Cefotaxime*) Immunostimulatory effects on neutrophil phagocytosis (*Ceftaroline*)	Reduction of nitrosative stress (*Ceftazidime*)	
**Polymyxins**	Stimulation *(Colistin)*	Enhance macrophages’ phagocytic function (*Colistin*) Innate immunity activation via a p38/PMK-1-pathway (*Colistin*)	Reduced NO production (*Polymyxin B*)	
**Fosfomycin**	Reduction of synthesis	Decreased the expression of TLR-4 and TNF in lung tissue during sepsis and ARDS		

Legenda: ROS = Reactive Oxygen Species; TLRs = Toll Like Receptors; NO = Nitric Oxide; LPS = Lipopolysaccharides; MMPs = Matrix Metalloproteinases; ALI = Acute Lung Injury; ARDS = acute respiratory distress syndrome; TNF = Tumour Necrosis Factor; LPS = Lipopolysaccharides.
